# Health care public reporting utilization – user clusters, web trails, and usage barriers on Germany’s public reporting portal *Weisse-Liste.de*

**DOI:** 10.1186/s12911-017-0440-6

**Published:** 2017-04-21

**Authors:** Christoph Pross, Lars-Henrik Averdunk, Josip Stjepanovic, Reinhard Busse, Alexander Geissler

**Affiliations:** 1 Dept. of Health Care Management, Berlin University of Technology, Administrative office H80, Str. des 17. Juni 135, 10623 Berlin, Germany; 2 Weisse Liste gGmbH, Leipziger Straße 124, 10117 Berlin, Germany; 3 European Observatory on Health Systems and Policies, WHO European Centre for Health Policy, Eurostation (Office 07C020), Place Victor Horta/Victor Hortaplein 40/10, 1060 Brussels, Belgium

**Keywords:** Public reporting, Quality transparency, Hospital quality, Provider benchmarking portal, Web usage mining, Cluster analysis, Markov chains, Clickstream analysis

## Abstract

**Background:**

Quality of care public reporting provides structural, process and outcome information to facilitate hospital choice and strengthen quality competition. Yet, evidence indicates that patients rarely use this information in their decision-making, due to limited awareness of the data and complex and conflicting information. While there is enthusiasm among policy makers for public reporting, clinicians and researchers doubt its overall impact. Almost no study has analyzed how users behave on public reporting portals, which information they seek out and when they abort their search.

**Methods:**

This study employs web-usage mining techniques on server log data of 17 million user actions from Germany’s premier provider transparency portal Weisse-Liste.de (*WL.de*) between 2012 and 2015. Postal code and ICD search requests facilitate identification of geographical and treatment area usage patterns. User clustering helps to identify user types based on parameters like session length, referrer and page topic visited. First-level markov chains illustrate common click paths and premature exits.

**Results:**

In 2015, the *WL.de Hospital Search* portal had 2,750 daily users, with 25% mobile traffic, a bounce rate of 38% and 48% of users examining hospital quality information. From 2013 to 2015, user traffic grew at 38% annually. On average users spent 7 min on the portal, with 7.4 clicks and 54 s between clicks. Users request information for many oncologic and orthopedic conditions, for which no process or outcome quality indicators are available. Ten distinct user types, with particular usage patterns and interests, are identified. In particular, the different types of professional and non-professional users need to be addressed differently to avoid high premature exit rates at several key steps in the information search and view process. Of all users, 37% enter hospital information correctly upon entry, while 47% require support in their hospital search.

**Conclusions:**

Several onsite and offsite improvement options are identified. Public reporting needs to be directed at the interests of its users, with more outcome quality information for oncology and orthopedics. Customized reporting can cater to the different needs and skill levels of professional and non-professional users. Search engine optimization and hospital quality advocacy can increase website traffic.

**Electronic supplementary material:**

The online version of this article (doi:10.1186/s12911-017-0440-6) contains supplementary material, which is available to authorized users.

## Background

Initiatives to measure and publicly report hospital quality have been implemented in many countries. They help to reduce information deficits and empower patients, their relatives, and payers to choose and contract with the most appropriate and highest quality providers. In particular, public reporting web portals are expanding rapidly in many OECD countries [1]. In the US, the CMS website *Hospital Compare* as well as several consumer reports, such as *Healthgrades.org* or *ConsumerReports.org*, provide quality of care information. In the UK, *MyNHS* and others enact the UK open data policy and the NHS quality transparency objectives. In Germany, the transparency portal *Weisse Liste.de (WL.de)* reports the results of the mandatory quality monitoring system. While *WL.de* is the leading German portal, other initiatives such as *Qualitätskliniken.de* also offer online quality of care information for participating hospitals. In total, Germany has eight portals that report hospital quality a national level and 10 portals that report quality a regional level [2].

Awareness for quality variation and treatment differences among patients is rising. In a recent representative consumer survey in Germany, half of respondents assumed that quality variation between hospitals is large [3]. In the US, a majority of people (55%) are dissatisfied with health care quality, and to compare hospital quality, they seek information on experience in certain procedures (65%), and mortality rates (57%) [4]. If information is well marketed and sought after, it appears to influence patients’ hospital decisions. An analysis of the influence of the regional Hospital Guide Rhine-Ruhr in Germany found a relative increase in patient market share for hospitals that report higher than average quality [5]. In the US, higher quality hospitals have been reported to have higher market shares and to further increase their market share over time [6]. Good hospitals have an inherent self-interest in reporting quality and stimulating quality competition, as competition in regulated health systems through other dimensions (i.e. price, staffing, location) is limited [7].

Yet, public reporting expansion and optimism among policy makers are contrary to doubts among researchers and practitioners about the actual impact of public reporting. A systematic review of 150 public reporting studies found that most public reporting tools face limited usage [1]. Overall, evidence for a positive effect of public reporting on consumer behavior or quality of care is limited, and public reporting often lacks impact on the behavior of health care professionals [8]. Reported outcomes are only one aspect influencing patients’ choice of hospital, with a variety of other hospital characteristics playing a substantial role as well [9]. In another US survey, only 7% of participants actually used hospital quality of care information to make health care decisions [10, 11]. In Germany, less than 20% of outpatient specialists are aware of public reporting websites and less than 10% use them actively for patient advise [12]. Causes for the often limited impact of public reporting include: complexity of quality measures, limited user-friendliness, lack of physician support and little integration into the care pathway, missing awareness of substantial quality difference between hospitals and thus motivation to search quality information and actually choose a good hospital, a mismatch between supplied and demanded information, and confusion about conflicting results on different websites for the same provider [1, 8, 13–19].

In general, studies examining health website user data are rare [1, 19], although analyzing web customer preferences is widely spread in other industries such as fashion retail and hospitality [20–22]. As the only study investigating traffic and user preferences for online public hospital quality information, Bardach et al. (2015) analyze website analytics data from a US group of hospital or physician public reporting websites and surveyed real-time visitors to these websites. Based on aggregated data (e.g. number of visitors, arrival method) and survey responses (type of respondent, purpose of visit, and website experience), they found that more than half of patients are willing to choose providers based on the information provided and health professionals generally have a better experience with public reporting than patients [23].

Past studies have been primarily based on smaller or regional patient or clinician surveys, examining changes to hospital case volumes based on reported information or only analyzed aggregated web usage data. To the best of our knowledge, there is no study that has examined in detail, based on large scale and detailed web usage data, how users actually behave on public reporting websites, which type of content they engage in, and where they abort their information search. Furthermore, most research on public reporting has been focused on a few countries, primarily the US, the UK and the Netherlands.

This paper aims to provide insights into the actual usage of online public reporting and identify public reporting improvement areas based on identified usage patterns. We first investigate whether information supplied matches patient demand and regional variations in public reporting usage. We then identify usage frequency and intensity of different portal sections and key user groups, their usage characteristics, and usage patterns. We use descriptive analyses and web mining techniques – web user clustering and first level Markov chains – on clickstream data from 17 million user actions from the *WL.de* hospital quality transparency portal from 2013 to 2015. At an overall level, we also contrast *WL.de* usage data with new and unpublished usage data from the *Hospital Compare* website.

## Methods

### *Weisse Liste* background

Annual, self-reported hospital report cards are compiled as part of the mandatory external quality monitoring system and gather structural information (such as case volumes, equipment, staff levels) across all medical specialties as well as process, outcome and risk-adjusted outcome quality indicators for 30 diseases and diagnoses, covering around 3.1 million cases or 15% of the annual case volume in Germany [24]. On behalf of several major statutory health insurance funds, the *WL.de* carries out the government mandate for the statutory health insurance (SHI) system to publically report the information in an easily accessible and patient friendly manner [25].

In 2008, the *WL.de* project was jointly initiated by the non-profit Bertelsmann Stiftung and the main patients’ and consumers’ associations. The portal *WL.de* has become the largest health care quality public reporting portal in Germany, consisting of a hospital, an outpatient physician and a nursing care search portal. The hospital search has gone through several development rounds, with the latest re-launch in June 2015. *WL.de* quality data is also integrated into websites of health insurance funds such as the AOK and the BARMER GEK.

**Fig. 1:**
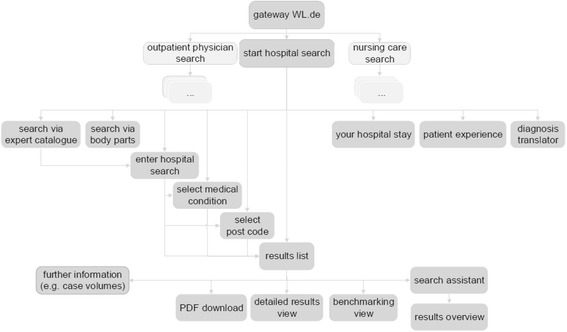
*WL.de Hospital Search* portal sitemap. Legend: Own design based on general website structure and functionality over data period from 2013–2015

Our analysis focuses on the *WL.de Hospital Search* portal (see sitemap in Fig. 1). Users search for hospital quality information by entering geographical (postcode or city) and disease, diagnosis or procedure information. Data is either entered directly or via assisted searches (via body parts or disease and procedure catalogues). Searches can be completed for 5–500 km radius or nationwide. After a valid geographical and medical information combination is entered, the website returns an initial results list of hospitals. The user can then either examine in detail a particular hospital (and generate a PDF report for the selected hospital) or initiate a benchmarking by selecting several hospitals for comparison. Other website elements present background information, current news and explanation on information relevance. The re-launch in mid-2015 has slightly changed the set-up and flow of the website compared to our observation period.

### Usage data

We received preprocessed server log files from the statistic module of the content management system Papaya CMS for 273 million server requests between 17.12.2012 and 28.05.2015 in a MySQL database dump file. We re-imported the server log files via MySQL 5.6, re-created a 100 GB MySQL database, and operated the MySQL database via MySQL Workbench. In addition, we also received cleaned web user session data from a second cookie-based tracking program (Piwik) for the period of April 2013 until April 2015, which we used to validate the cleaned Papaya CMS data.

We completed extensive data cleaning as the website is highly frequented by robots originating from search engines indexing as well as from fraudulent data siphoning. Search engine robots, with a share of 57% of all raw log file entries, are easily identified and excluded. Masked, fraudulent robots must be detected manually by rules-based cleaning. Furthermore, we also excluded traffic generated by non-human sources, e.g. Ajax-requests and requests originating from RSS-Readers. After data cleaning, 17 million user server requests remained. In order to analyze user behavior, we reconstructed individual user sessions from the SQL server log files using established web usage mining methods [26, 27]. An example of the individual SQL server requests and the associated user sessions are displayed in Additional file 1.

The WL.de website user data is proprietary and WL.de competitive concerns as well as data usage restrictions within the public quality monitoring system do not allow data sharing beyond the limited and vetted circle of the author team. Specifically, the WL.de data privacy stipulations as well as the licensing agreement between WL.de and the SHI funds explicitly disallow data passage to external parties outside the influence of WL.de [28]. Moreover, a data usage agreement between WL.de and the author team was signed that limits the usage of this data to the scientific purposes of this study.

### Methodology

In online consumer research, clickstreams (i.e. web user trails or navigational patterns) take an increasingly important role in helping marketing professionals and researchers to uncover online consumer behavior based on large scale online shopping data. More precisely, the term “clickstream” denotes the electronic record of a user’s activity through one or more web sites and reflects a series of choices in navigating the web [20, 29, 30]. We first investigated the clickstream user session data at a more general level with descriptive methodologies for the entire data period from mid-December 2012 to end of May 2015. Afterwards, we employed e-commerce web usage mining techniques to infer detailed user patterns, usage barriers and user information gain and model user trails, or a sequence of web pages viewed by a user in a certain timeframe [31, 32]. We limited the time period for the detailed clickstream analyses to the first five months in 2015 to choose a distinct, comparable and recent time period where the site structure has not changed and to circumvent computational limitations. For the detailed analyses, we also exclude bounce visits – when users leave immediately after entering the portal [33]– and visits to the AOK and BARMER WL.de sub-portals.

The aim of the cluster analysis is to identify user groups according to their usage behavior and interests, which is captured in their clickstreams. Knowledge of the different user types, or communities, can facilitate the improvement of the website by identifying and satisfying differentiated user needs and preferences [34, 35]. For our clustering, we include two types of user information, displayed in Table 1. On the one hand, usage specific clickstream variables such as clicks per session, time of access, etc. are included. On the other hand, content related variables are taken into account. Therefore, a methodology to cluster mixed data is required to incorporate the different types of variables. To determine similarities and dissimilarities between the sessions, the Gower’s General Similarity Coefficient is used as distance measure [36]. The advantage of this measure, compared to the more frequently used Euclidean distance measure, is that Gower’s distance measure works with mixed data types and avoids an aggregation of variables and associated information loss [37].

**Table 1:** Clickstream variables and information content for clustering

Variable	Type^1^	Mean	SD	Description
Number of clicks	cont	13	15	User click on website element (request)
Time per click	cont	600	909	Time in seconds passed between clicks
Successful visit	cat	68%		Success = view of hospital search results
Work time access	cat	51%		weekdays 9.00 am - 6.00 pm
Mobile device	cat	17%		Use of handheld device
Returning	cat	22%		Returning visitor with previous visit
Referrer				Webpage where the user came from
Direct entry	cat	21%		*WL.de* directly entered in URL bar
Search engine	cat	68%		*WL.de* entered via search engine (e.g. google)
Health magazines	cat	4%		Patient health magazines (e.g. Apothekenumschau)
Health insurance	cat	3%		Statutory health insurance websites
Media	cat	2%		Online news sites
Internal link	cat	2%		Other *WL.de* portals (e.g. nursing care)
Other	cat	1%		
Website content				Content visited by average user (clicks per topic)
Start hospital search	cont	10%	15%	Initiate search based on medical and geograph. info
Select medical condition	cont	5%	12%	
Search via body parts	cont	3%	11%	Select medical condition via human body part map
Search via catalogue	cont	1%	7%	Select medical condition via ICD/OPS^2^ expert list
Select post code	cont	1%	3%	
Search results	cont	23%	24%	List of hospitals offering relevant care in geo area
Detailed results view	cont	13%	23%	Detailed information about one selected hospital
Benchmarking	cont	1%	4%	Direct comparison for selected criteria/hospitals
PDF brochure download	cont	0%	1%	Download info about selected hospital(s)
Diagnosis translator	cont	11%	31%	Find medical descriptions for ICD/OPS^2^ codes
Your hospital stay	cont	1%	5%	
Patient experience	cont	0%	3%	Information about patient experience survey
Background info	cont	0%	3%	Background info about *WL.de* transparency project
Latest news	cont	0%	2%	
Sister sites	cont	10%	21%	Information on outpatient physicians, nursing care

Clickstreams analyses based on 80,000 session data sample from January to May 2015 excluding bounce visits. Clustering was conducted based on 22 variables (referrer functioning as one variable); mean and standard deviation calculated for the average session in the sample

1. Variable continuous or categorical (dummy 1 = yes, 0 = no); 2. ICD = International Classification of Diseases, OPS = Operationen- und Prozedurenschlüssel (based on International Classification of Procedures in Medicine)

We apply a hierarchical clustering algorithm, which is more resource intensive than the often used k-means algorithm but allows retrospective determination of cluster quantity based on stopping rules such as the Duda-Hart-Index [38] and graphical interpretation of dendrograms. Among several possible hierarchical clustering algorithms, we choose Ward’s minimum variance method as it is for the data structure fitting algorithm and capable of identifying consistent, actual user groups [37, 39–41]. Other algorithms such as the single-linkage and complete-linkage algorithms were tested and ruled out due to high outlier and data noise sensitivity [42].

To visualize navigation paths, we employ Markov chain modelling, which regards each website content area (Table 1) as a separate state and links between the topics as transitions [43]. The model contains the transition probabilities from one website topic area to another [42]. We use first-order Markov chains, where the probabilities for the next visited site depend only on the previously visited site [44]. To ensure stability of results, we ran the clustering algorithms and the Markov chain clickstream analysis multiple times for many different data samples from the first half of 2015 observation period. We also challenged the clustering and clickstream results in several workshops with different *WL.de* experts.

All analyses are conducted at an aggregated or large group level, with no individual or small user group identification. The server log files include no data privacy sensitive information. IP addresses were anonymized and used only to track returning visitors. To get access to the web portal user data, the proposed analyses and methodology were vetted by WL.de in consultation with its SHI stakeholders and found to comply with the stringent data privacy concerns [28]. Thus, our methodology and data use comply with the relevant ethical stipulation and no other approval of an additional ethics committee is required.

## Results

### Overall usage pattern

On average, *WL.de* has 10,000 daily users, of which 30% search for hospital quality information. Unique website visits per day have increased from 1,445 in 2013 to 2,753 in 2015 (Table 2), which is an annual compound increase of 38%. In 2015, daily visits added up to 980,000 annual visits. Visits per 1,000 hospital admissions have almost doubled, from 28 in 2013 to 52 in 2015. In 2015, on average, visitors spent 6.7 min (399 s) per visit and conducted 7.4 clicks, with 54 s per click. In 2013, visitors spent substantially more time on the portal (9.4 min), conducting more clicks (10.8 clicks per visit), but taking slightly less time between clicks (52 s). Overall, time spent on the portal has decreased by 30% from 2013 to 2015. The bounce rate has increased from 22% in 2013 to 38% in 2015, which is a 73% increase and in line with increased mobile usage (11% in 2013 and 25% in 2015). During the observation period, the *WL.de* portal was not mobile-responsive, while mobile information search on *WL.de* and the general internet increased substantially [45]. The increased bounce rate can be at least partially attributed to the higher share of mobile users, which have higher bounce rates and non-successful visits (no results information).

**Table 2:** Summary website traffic for WL.de and Hospital Compare hospital search 2013–2015

	Weisse Liste.de			Hospital Compare		
Variables	2013	2014	2015	2013	2014	2015
Unique visits per day	1,445	2,122	2,753	3,476	3,072	3,806
*Growth p.a. [%]*	-	47	30	-	−12	24
*Visits per 1,000 hospital adm.*	28	40	52	35	32	40
Clicks per visit	10.8	8.4	7.4	3.4	4.0	3.8
Time per visit [sec]	566	456	399	91	89	92
Time per click [sec]	52	54	54	-	-	-
Bounce visits [%]	22	32	38	37	32	34
Successfull visits [%]	66	53	48	-	-	-
Mobile visits [%]	11	23	25	-	-	-
Referred via Google search [%]	23	42	38	-	-	-
Referred via Google adWords [%]	0	0	14	-	-	-
Entered directly [%]	35	27	24	-	-	-

WL.de data from Q1 for each year 2013, 2014 and 2015 since data for those quarters most complete and comparable across the three years; data comparability between WL.de and Hospital Compare to the best of our knowledge; data including bounce visits

To put the *WL.de* results into perspective, we received US CMS data on overall usage for the *Hospital Compare* portal (Table 1), where daily visits increased from 3,476 in 2013 to 3,806 visits in 2015. While absolutely still lower, usage at the *WL.de* hospital search has increased more rapidly between 2013 and 2015 than for *Hospital Compare*. Weighted by the number of hospital admissions, relative *WL.de* hospital search usage has surpassed *Hospital Compare* usage. However, in the US, many more national websites exist that report hospital quality information. In particular, *Healthgrades.com* is more commonly searched for than *Hospital Compare* [46], which implies an overall higher public reporting usage in the US than in Germany. Bounce rates for both websites are roughly equivalent and within the range of acceptable bounce rates [47]. Both clicks per visit and average time per visit are substantially longer at *WL.de*, which can be explained by the different public reporting approaches. *WL.de* reports at the medical condition, hospital and single quality indicator level (requiring more time to make the relevant selections), whereas Hospital Compare reports more generally at the aggregate hospital level, with composite information across medical conditions.

The share of hospital search users entering the website via the Google search engine has increased from 23% in 2013 to 38% in 2015 while the Google AdWords has increased from 0% in 2013 to 14% in 2015. In total, the number of daily users arriving through the Google search engine (market share of 95% in Germany) has increased from 890 in 2013 to 1,430 in 2015, which is an increase of 60% and three times the increase of use of internet search engines for consumer information search [48]. Accordingly, the share of users entering the website directly has decreased from 35% in 2013 to 24% in 2015. As a key performance indicator, the share of successful visits – users viewing hospital search results – has decreased from 66% in 2013 to 48% in 2015, which can likely be attributed to the higher share of mobile visits, as the website was not mobile responsive.

**Fig. 2:**
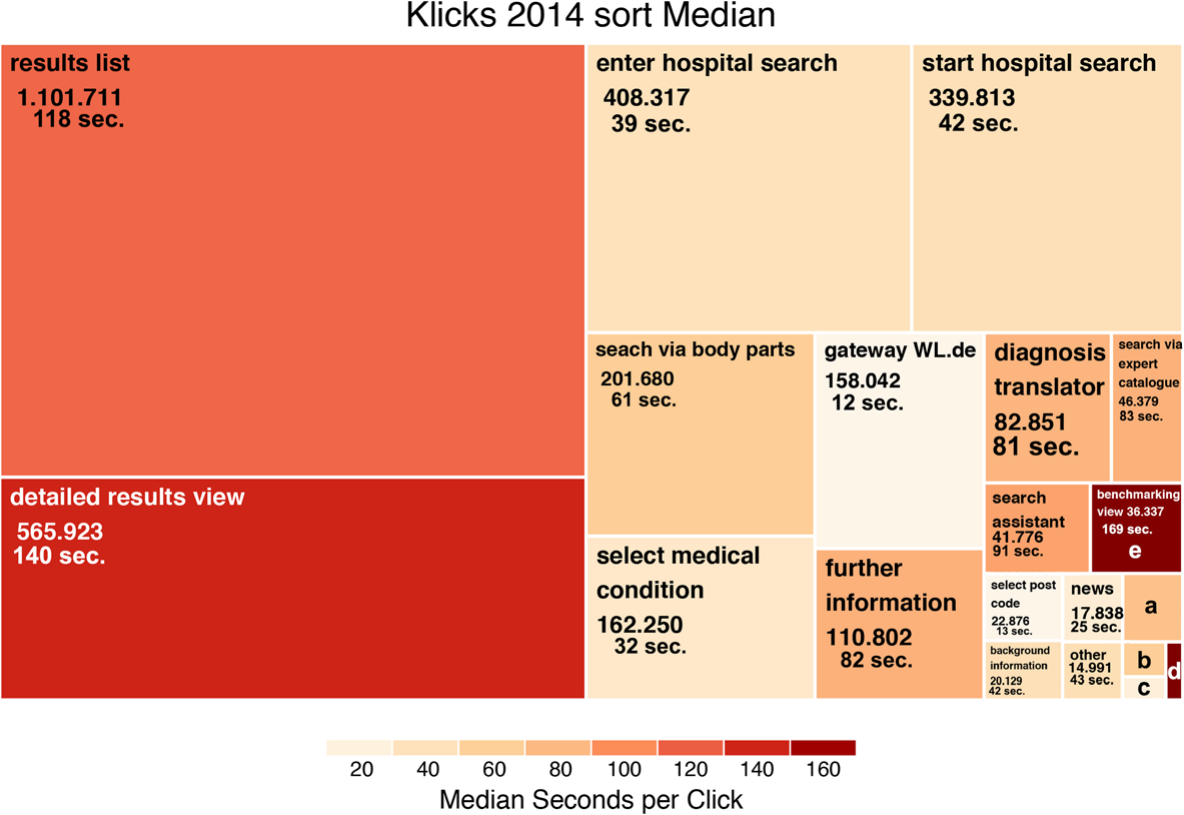
Heat map displaying number of clicks and usage intensity, fully year 2014. Legend: Own calculation. Size of rectangles captures number of clicks in topic area and color code displays average time spent in topic area. Information calculated based on 2014 data (to increase sample size). Robustness checks for full year data 2013 and 5 months’ data from 2015 provide consistent results

The heat map for the most recent and full year 2014 in Fig. 2 summarizes website usage based on visited website elements (topic areas). Users conduct most clicks on the initial results lists (1.1 million clicks), followed by detailed results for one hospital (0.6 million) and the hospital search entry mask (0.4 million). The initial results window is the most popular way of viewing results while the detailed and benchmarking view options are substantially less frequented. In contrast, users spent most time on the benchmarking window (170 s), followed by the detailed search results (140 s) and the results window (118 s). Once users have reached the more detailed search results, they take a longer, more detailed look.

### Hospital quality information supplied vs. demanded

To investigate the fit between publicly reported (i.e. supplied) hospital quality information and patient demand, we determine the top 20 requested medical conditions on *WL.de* and contrast those with the outcome quality information collected and reported within the mandatory quality monitoring program (Table 3). The ranking is based on the number of user requests weighted by the 2014 disease incidence. The two primary diagnostic groups for which users want to compare hospitals are cancer and orthopedics, contributing 10 and 5 search terms to the top 20 diagnoses, respectively. While these 15 diagnoses generate substantial patient interest in public reporting of hospital quality, process or outcome quality of care indicators are only available for two orthopedic (arthrosis in knee or hip) and two cancer diagnoses (breast- and ovarian cancer). For highly demanded diagnoses, such as prostate, esophageal and colon cancer or spinal disc herniation, internal derangement of knee, and depression, no process or outcome quality indicators are available on *WL.de* or the mandatory quality reporting program.

**Table 3:**
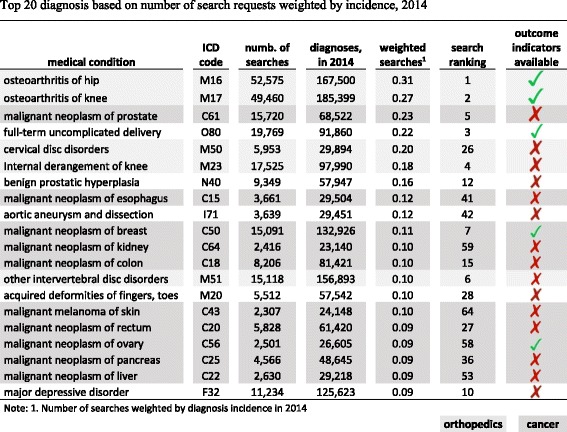
Top 20 diagnosis based on number of search requests weighted by incidence, 2014

### Geographical usage patterns

**Fig. 3:**
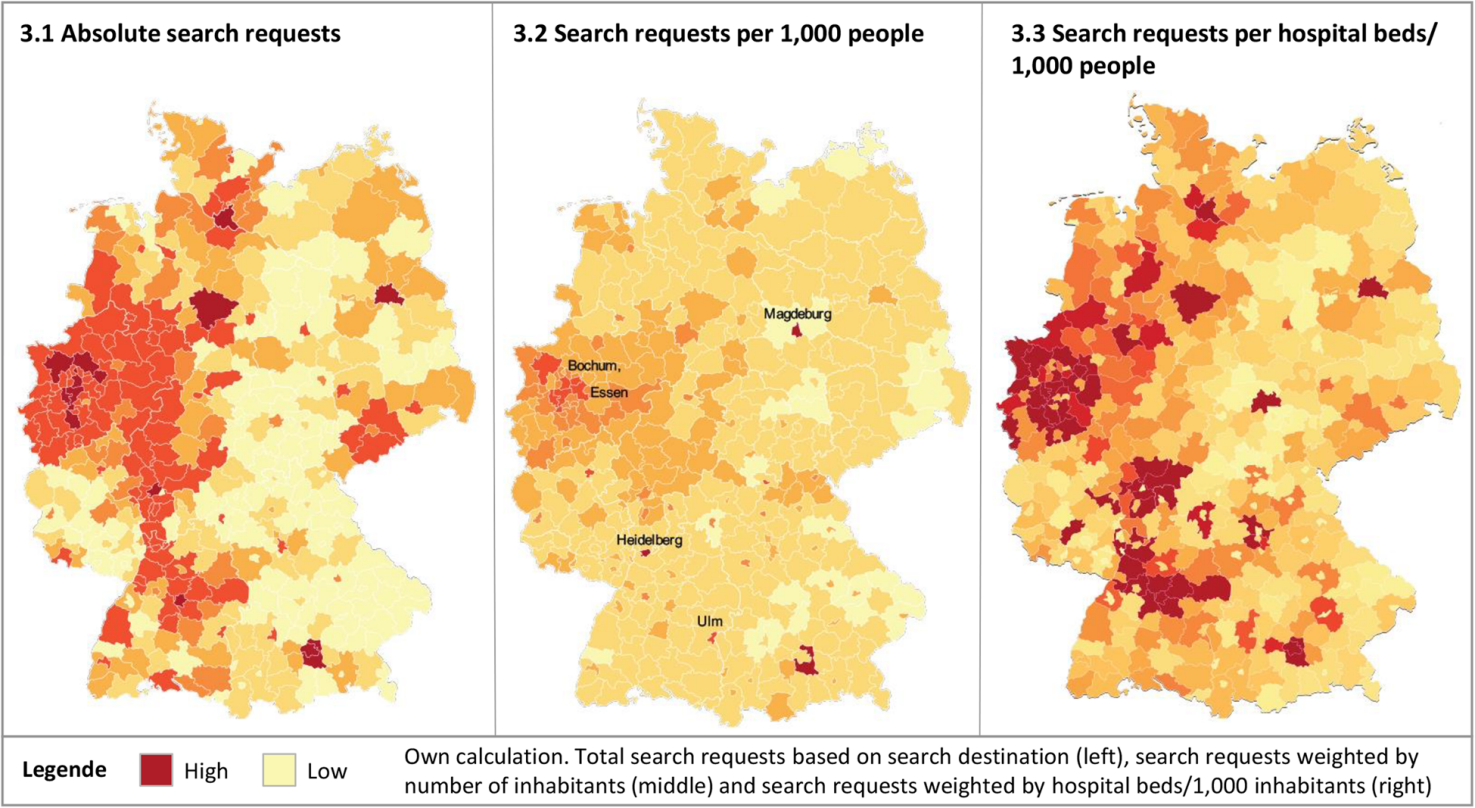
District-level maps of Germany displaying *WL.de* usage. Legend: Own calculation. Total search requests based on search destination (left), search requests weighted by number of inhabitants (middle) and search requests weighted by hospital beds/1,000 inhabitants (right)

Figure 3 shows regional variation in public reporting usage patterns, with raw usage figures on the left. Due to population density, usage (based on search destination) is highest in the metropolitan areas Berlin, Munich and Hamburg and the state of North Rhine-Westphalia in West. When adjusting for number of inhabitants (middle), usage becomes more evenly distributed. However, medium-sized cities with medical universities, e.g. Magdeburg, Heidelberg und Ulm, have higher than average usage due to large catchment areas. Likewise, North Rhine-Westphalia shows higher search activities than other parts of Germany even after adjusting for population density. When adjusting for hospital capacity in each district (number of hospital beds per 1,000 inhabitants) (on the right), urban areas with lots of hospital beds stand out.

### User clustering

Results of the clustering algorithm show ten distinct user types (Table 4). These user types address similar character differentiations as previous user community studies in a health information setting [23, 49] and other e-commerce web trail studies [50, 51]. The two largest groups are the *Intensive Work Time* users (19% user share), which have 100% work time usage and a higher share of returning visitors, and the *Intensive Free Time* users (17%), which have 0% work time usage and have a small share of returning visitors. Both groups view hospital quality results in 100% of cases. On average, both groups spent 11–12 min on the website and conduct 15–16 clicks per visit. Both groups also have 100% desktop usage and all enter the *WL.de Hospital Search* via search engines.

**Table 4:** User cluster and their usage characteristics

User cluster	Share [%]	Average number clicks^2^	Average visit length [sec]^2^	Time betw. clicks [sec]^3^	Return visitors [%]	Viewed results [%]	Search steps/results^1^	Visit dur. Workday [%]	Working hours [%]	Desktop usage [%]	Access via [%]
Intensive Work Timers	19	15.2	693	45	29	100	0.45	100	100	100	100 search engine
Intensive Free Timers	17	16.4	731	46	13	100	0.42	43	0	100	100 search engine
Diagnosis Translator	13	5.7	182	32	19	-	-	100	100	100	100 search engine
Challenged Aborts	12	6.1	255	48	8	-	-	53	12	69	100 search engine
Patient Experts	9	16.7	851	54	24	100	0.33	63	12	66	100 direct
Curious	7	14.9	747	49	28	78	0.60	83	53	83	35 payer, 30 media
Professionals	7	15.9	884	53	56	100	0.32	100	100	100	100 direct
Results Mobiles	7	14.5	696	49	8	100	0.50	65	30	0	100 search engine
Explorers	4	13.4	571	47	14	72	0.67	77	48	80	100 health website
Other	5	6.5	456	72	40		-	79	42	74	100 direct
Average User	100	12.7	596	47	22	68	0.42	76	51	83	67 search engine

Clustering based on clickstream data and repeated sampling from data sample from 01/2015 – 05/2015 excluding bounce visits

1. Search steps required relative to number of results viewed; 2. all clicks or visit lengths in sec per user cluster/number of users in cluster (weighted average); 3. Calculated per session and then averaged for user cluster (simple, non-weighted average)

The third largest group is the *Diagnosis Translators* (13%), which, on average, only spend 3.2 min during working hours on the website and do not view any results, but instead translate their ICD diagnosis or OPS procedure code into understandable descriptions, with a 20% share of returning users. No one of the *Diagnosis Translators* is using the hospital search function for the inquired medical condition or respective postal codes.

The fourth largest group, the *Challenged Aborts* (12%) abandon their search after only 6 clicks and 4 min on the website, without viewing any results information. All enter through search engines, one third uses a mobile device and most access the page during non-working hours. Furthermore, the *Patient Experts* (9%) access the portal directly, mostly after hours during the week. One third uses a mobile device and two thirds their desktop computers. They have the highest number of clicks (17), spent almost 16 min on the page, have a higher share of returning visitors and all of them view hospital results. Similarly, the *Professionals* (7%) spend more than 16 min on the website, conduct 16 clicks and view results in 100% of visits, but access the portal 100% during working hours and 100% through their desktop machines. Importantly, 100% of *Professionals* access the website directly and more than half of them are returning visitors (highest share of all user types).

### Clickstream analysis

**Fig. 4:**
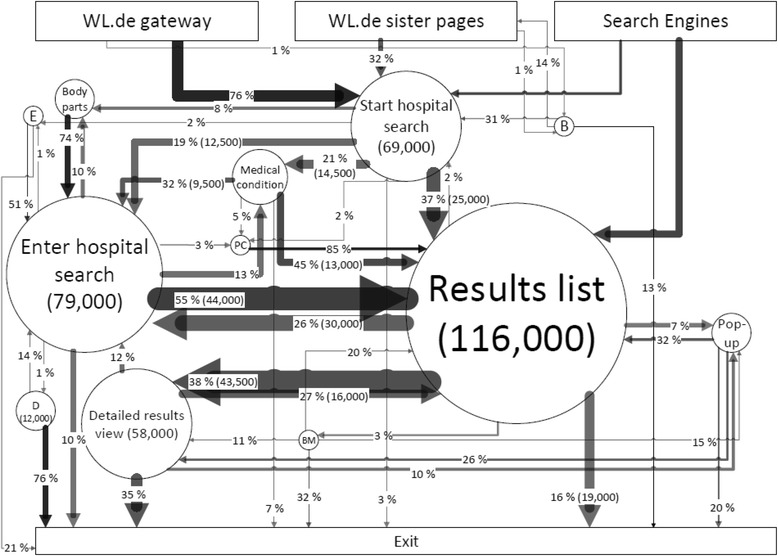
Overall navigation trails for all users. Legend: bubble size = clicks per topic area, arrow width = absolute number of transitions for entire page, arrow grayscale = share of transitions away from topic area (i.e. bubble), B = background information, BM = benchmarking view, D = diagnosis translator, PC = select post code, E = expert catalogue (ICD/OPS lists), Pop-up = detail information pop-up

Figure 4 displays the overall navigation trails for the *WL.de Hospital Search* portal. Most users access the hospital search portal via the *WL.de* gateway. From the hospital search entry page, 37% of users enter hospital search information correctly and go directly to the hospital results list. 48% of users require support in their hospital search, with 21% selecting a medical condition based on a drop down list, 8% via body parts display and 19% hospital search window. From the hospital search entry page, 55% of users complete the search correctly and arrive at the initial results view. 13% have to reselect a medical condition, 10% return to the body parts display and 3% re-select a post code. 10% of users exit prematurely from the hospital search entry page. When viewing the initial hospital results, 48% of users click on a specific hospital to view detailed results, 26% return to the hospital search page to change their search parameters or conduct a new search, 16% exit without viewing more detailed results, 7% of users look for more detailed explanations via the info popups and only 3% actually navigate to the benchmarking function.

A large share of users of the diagnosis translator function (76%) exit without searching for hospital quality results (i.e. the *Diagnosis Translators*). Likewise, a significant share of users exits directly from pages with additional information, such as background info (13%) and info popups (20%). Furthermore, a considerable share of users exits during the assisted search process (10% from the hospital search entry page, 9% while using the body parts display and 7% while selecting a medical condition from the drop down.

Combining the user cluster and clickstream methodology, Additional file 2: Figure S1, Additional file 3: Figure S2, Additional file 4: Figure S3, Additional file 5: Figure S4 in the Additional files separately display the clickstreams for important user clusters. The *Intensive Work Timers* as the largest cluster display a similar navigation pattern as the patterns described above for the average user. However, the *Patient Experts* as well as the *Professionals* use less frequently the assisted search functionalities. The *Challenged Aborts* display a very erratic navigational pattern and often return to a previous node, exit often from the assisted search function, the hospital search entry page, and the additional information pages, and often return to the *WL.de* gateway or sister pages without completing a hospital search.

## Discussion

*WL.de Hospital Search* usage has increased substantially, up to 2,753 daily users in 2015. Compared to 2013, users have spent less time on the webpage and more frequently not requested any hospital quality information. Relative to *Hospital Compare*, *WL.de* usage has shown a stronger growth in usage. However, since the US has several other equally or even more popular public reporting sites such as *Healthgrades.com* public reporting experiences higher usage in the US. But public reporting usage in Germany is catching up. The *WL.de* traffic growth far outpaced the overall growth of internet users in Germany [45]. As illustrated by the heat map, the more *WL.de* detailed results formats (individual hospital details view or benchmarking view) receive substantially fewer visits (only 51% and 3% relative to the 1.1 million clicks on the results page, respectively), but usage intensity is substantially higher (19% and 43% more time relative to the 118 s on the results view, respectively).

The demand vs. supply analysis has revealed a gap between hospital quality information demanded by patients on WL.de and quality indicator information provided by the quality monitoring system (Table 3). The most-searched-for diagnostic categories, for which outcome quality information is missing, are prostate, esophageal and colon cancer and the orthopedic diagnoses spinal disc herniation and internal derangement of knee, as well as depressive disorders. The lack of relevant outcome information can hinder the acceptance of public reporting as users do not find information they are looking for. Comparing usage across geographic areas, people living in Western German regions, especially the state North-Rhine-Westphalia, show a particular affinity for public reporting. One contributing factor could be the higher awareness of public reporting and the quality difference between providers, due to regular publication of the Hospital Guide Rhine-Ruhr [5, 52], which is one of the earliest public reporting products target at the general public. Another contributing factor could be higher hospital density and thus more choice relative to other states [53].

The different cluster and click chains illustrate substantial variation in user interests and behaviors, indicating both the need to provide flexibility in information access, type and detail and overall improvement potential for public reporting. On the one hand, a substantial share of users does not view any hospital results information (32%) and, on the other hand, many users do not view more detailed and possibly more informative benchmarking or detailed single hospital information. Referrer and amount of time spent on the webpage as well as interest in background and explanatory information vary among clusters.

Public reporting is supposed to encourage patients to choose high quality providers. Provider selection is also what fuels quality competition among providers and drives improvements through changes in care [54]. Since public reporting should be the basis of provider selection and the quality improvement pathway, ineffective public reporting has important consequences. Optimizing public reporting has two primary elements. *Onsite*, the right content needs to be presented in the best format and detail level for different user groups and their navigation patterns. *Offsite*, web traffic management needs to be optimized to ensure maximum traffic via search engine optimization and increased awareness of the benefits and functionality of public reporting via media communication and expert commentary.

### Onsite

The cluster analysis illustrates different usage patterns and interests for the various user groups. Different user demographics and purposes require different types and detail levels of information. For example, elderly patients or those with lower levels of education generally have more difficulty in understanding comparative health information [55, 56] and thus have distinct information needs. Certain patient groups, such as younger, highly educated, or higher income patients or patients without previous satisfactory provider interaction, have been found to search more actively for a provider [8]. While the web analytics data does not provide demographic information, a separate, 2015 *WL.de* onsite user survey sheds light on user demographics. One third of *WL.de* users are above 60 years of age and another third between 50 and 60. Next to professional and personal use, 25% of users help family members in their hospital choice. A large share of users (42%) came to the portal not having chosen a provider yet.

A site that is flexible to adapt to these differences is more likely to provide information that users want [57]. The *WL.de* portal already is an interactive website that allows personalized searches based on user background (geographical and medical information). But public reporting needs to provide more flexible and customizable search and output displays to allow different user types to navigate the page and information based on their preferences and skills levels.

An important user differentiation is the professional (outpatient physicians, health advisor at insurance funds, patient advocates) vs. patient perspective. Our clustering results show that at least 7% of users can be classified as *Professionals*. In addition, a large share of users in the *Intensive Work Time* (19%) and *Diagnosis Translators* (13%) groups also have professional backgrounds. In a *WL.de* onsite survey, 24% of users identify themselves as professional users. Professionals and patients have different requirements for technical vs. non-technical information and presentation types. Even among professional users, different technical backgrounds and the ability to take in, process, and communicate information exist. Finding the right way to address *Professionals* is critical for public reporting, as admitting physicians play a large role in patients’ hospital choices, but still harbor substantial skepticism and resistance towards public reporting.

Specialists often question the credibility and usefulness of outcome data [58]. Similarly, general physicians often have a negative view of public reporting, primarily due to risks of insufficient risk-adjustments, oversimplification and patient skimming by providers [59]. Public reporting usage among specialists is limited [12]. The *WL.de* portal currently has no feature to separately address expert physician users, e.g. in tailored micro site. However, if public reporting differentiates more thoroughly between professional and non-professional users, information search, display, cognitive aids, interpretation and transfer can be more customized [23].

More customized or even personalized websites could streamline and ease the information search process for physicians, but also for patients, as returning visitors will often search for similar information (e.g. same geographic area). This information can be preselected in their personalized profiles (accessible via login). More generally, three hospital search entry buttons for new and experienced patient and professional users and customized search paths, information display and detail level can create customized public reporting.

The individual value of public reporting can be approximated by user behavior, e.g. whether the information is considered superficially or in detail. Our results show that few users navigate to the detailed result view options (detail or benchmarking view), but these website areas experience the most intensive engagement. Furthermore, 560 daily users abort the search before viewing hospital quality results, exiting prematurely from website elements such as the search function or background information.

Research consistently finds that in complex and uncertainty decision environments, consumers often make better evaluations and decisions when they are presented with less information and options about their choices. Furthermore, across display-response studies in the relevant health care literature, numerical formats that included extensive text were generally less effective than simple, more visual formats such as graphs or familiar icons [56]. Limiting consumers’ choice menu to the most relevant options, via geographical filtering or additional filters such as a predetermined quality filter, can support active decision making. Likewise, ranking information can improve comprehension, particular with older patients, make options easier to assess and reduce faulty data interpretations. In general, public reporting has many applications to using nudges to guide better decision making [14].

More broadly, if consumers have a general understanding of the overall paradigm (i.e. quality difference between hospitals), they will more likely understand smaller pieces of information and integrate them into their decision process [60]. Consumers in health care lack an understanding of what a choice might actually mean, once the decision is carried out [61]. Getting patients to form awareness of the benefits of active hospital choice and choice preferences prior to their actual choice helps to simplify and improve choice processes [62]. This implies that public reporting also has a role in generating more general awareness of quality variation between hospitals and benefits of hospital choice.

### Offsite

When examining public reporting optimization potential offsite, the three primary levers are search engine optimization, expert content placement and user-orientation of quality measurement. In 2013 and 2014, *WL.de* portal was optimized for search engines (on- and off page), which increased the share of Google referrers from 23% in 2013 to 38% in 2015 (Table 2). In particular, website URLs were made more distinguishable (e.g. by including hospital names) and website metadata, which Google uses for search referencing, was individualized, by e.g. changing the reference from “Weisse Liste hospital search – detail profile” to “[hospital name] in Berlin – Weisse Liste”. Tagging specific hospital names increases hit rate and relevance for users and overall traffic. Additionally, at the end of 2014 *WL.de* started to use Google Grants, the non-profit edition of Google AdWords, to advertise its hospital search. This led to a substantial share of users clicking on sponsored links – combining the terms hospital search and the requested city – at the top of the search results (14% in 2015). This also allows regional targeting to potentially increase public reporting in, e.g., areas where hospitals are possibly consistently underperforming or public reporting usage is low.

Public reporting websites can also increase their traffic via promoting expert content placement and associated media messaging. In November and December 2014, a regional German television station, the Hessian Broadcasting Corporation, ran a *WL.de*-supported program on quality in five large Hessian hospitals, such as the University Hospital Frankfurt. A central part of the program was *WL.de* quality data, which was explained by a *WL.de* expert. Furthermore, a short film promoting the *WL.de* hospital search was shown. During the first two weeks of the programming, *WL.de Hospital Search* traffic was 30% higher (3,365 visits per day on average) than during the two weeks before the first show on November 19^th^ 2014. Similarly, the AOK-Hospital Report 2014, released on January 21^st^ 2015, included an article about substantial medical errors in Germany. The extensive media attention also covered the AOK and *WL.de* hospital quality search portals and led to a usage spike, with 50% increased daily average traffic in two weeks after publication.

Orienting mandatory quality measurement schemes more towards the medical conditions and information users are actually searching for also increases relevance and usage of public reporting portals. Currently, patients search hospital quality information for many medical conditions for which no outcome quality indicators are available. Less than 30% of inpatient care is covered by the mandatory quality reporting [2, 63] and outcome quality information for many highly sought after oncological and orthopedic conditions are missing. Like any other industry, health care public reporting needs to identify and primarily address the needs of patients as the customers of health care provision.

### Limitations

With regards to data and methodology, we consider some shortcomings. Server log-based user tracking, as opposed to cookie-based user tracking, relies on user IP addresses, which can change due to rooter re-start or service provider maintenance. Servers can also fail to account for requests that are cached by the users’ computer or proxy servers or information might be lost in communication with the client [20]. The return user tracking had to be completed manually, as the automatic user tracking via Papaya was not activated while the log files were saved. However, comparison between our server-log-based user tracking and the Piwik cookie-based user tracking showed high consistency. Analyzing web usage data often faces the challenge of changing web-site structure and content; however, for the more detailed clustering and clickstream analysis we consider a narrower timeframe with no major structural changes and we use web design predefined topic categories that remain consistent even if content within these topic changes.

As a general methodological limitation, our approach of using clickstream data (as opposed to user survey data or experiments) does not allow a clear view on what users do after they leave the webpage, like whether they actually use the information to make a decision. Furthermore, we cannot deduce what users feel or experience while using the webpage. Combining clickstream with survey response data from the same users might serve as a solution here. High dimensionality clustering (in our case 22 variables) can at times provide non-logical, impractical results; however, we verified the clustering by confirming a priori hypotheses on typical user characteristics with the revealed characteristics of our user groups and extensive discussion of user group characteristics with multiple *WL.de* experts.

## Conclusions

Presenting public reporting information in a way that is most accessible for users can help to enhance the role of quality of care in treatment and hospital decisions, leading to better outcomes for patients. Public reporting promises to affect health care markets through the individual and collective informed choice of health care consumers. However, non-professionals often find it difficult to utilize quality data as information is often complex and the decisions carry high risks. Therefore, patients seek easily accessible and understandable information to make informed choices. For public reporting to realize its promise, further efforts need to be undertaken to provide context on the need of and motivation for quality of care information usage, simplify and enhance reporting portals; provide flexible, customized or even personalized usage options; offer quality information that is demanded by users; and embed quality of care information in the treatment pathway. This is especially true, since, compared with other consumer choices, health care and hospital choice decisions are complex and involve a high degree of uncertainty.

Additional research is needed to understand large sample, actual web user response to different information displays, content and detail levels. Compartmentalizing public reporting websites and monitoring user response to design and content changes can deliver real world data on what works best to engage users and facilitate their hospital choice and professional recommendations.

## Additional files

Additional file 1: Data example. This supplementary material includes raw data SQL requests for two user sessions and the associated user sessions that were created based on the raw data. A short data explanation describes the data and how it was used to create user sessions. (DOCX 177 kb)

Additional file 2: Figure S1. Navigational trail for user group Intensive Work Timers (19% of users). The figure depicts the navigation trail for the specific user subgroup Intensive Work Timers, indicating clicks per topics area (bubble size), absolute number of transitions (arrow width) and share of transitions away from respective topic areas (arrow grayscale). (TIF 875 kb)

Additional file 3: Figure S2. Navigational trail for user group Patient Experts (9% of users)**.** The figure depicts the navigation trail for the specific user subgroup Patient Experts, indicating clicks per topics area (bubble size), absolute number of transitions (arrow width) and share of transitions away from respective topic areas (arrow grayscale). (TIF 726 kb)

Additional file 4: Figure S3. Navigational trail for user group Professionals (7% of users). The figure depicts the navigation trail for the specific user subgroup Professionals, indicating clicks per topics area (bubble size), absolute number of transitions (arrow width) and share of transitions away from respective topic areas (arrow grayscale). (TIF 86 kb)

Additional file 5: Figure S4. Navigational trail for user group Challenged Aborts (12% of users). Description: The figure depicts the navigation trail for the specific user subgroup Challenged Aborts, indicating clicks per topics area (bubble size), absolute number of transitions (arrow width) and share of transitions away from respective topic areas (arrow grayscale). (TIF 784 kb)

## Abbreviations

AOK: Allgemeine Ortskrankenkasse
CMS: Centers for medicare and medicaid services
ICD: International classification of diseases
OPS: Operationen- und Prozedurenschlüssel
Papaya CMS: Papaya content management system
RSS-Readers: Really-simply-syndication reader
UK: United Kingdom
US: United States of America
WL.de: Weisse Liste.de
