# Supplementary material for: Health care public reporting utilization – user clusters, web trails, and usage barriers on Germany’s public reporting portal Weisse-Liste.de
Source: BMC Med Inform Decis Mak. 2017 Apr 21;17:48. doi: 10.1186/s12911-017-0440-6 (PMC5399803; doi:10.1186/s12911-017-0440-6)
Supplement: Supplementary file 1 — Data example. This supplementary material includes raw data SQL requests for two user sessions and the associated user sessions that were created based on the raw data. A short data explanation describes the data and how it was used to create user sessions. (DOCX 177 kb) [file 12911_2017_440_MOESM1_ESM.docx]

**Supplementary Material 5 – Data Example**

**Short explanation:**

The SQL request log files represent the raw data. Based on the IP address, the user agent and the country we created unique user IDs. The URL of each request (statistic_uri) and the respective filename and topic_id describe the content the user engaged with. We then aggregated the sequential requests to sessions, which represent the full website visit of the respective user. Based on the raw data, we also derived several variables (e.g. device type or referrer category) to characterize the sessions. All analyses in the article were carried out on a session level.

**User session example**

**SQL request example**
