# Supplementary figures and images for: Health care public reporting utilization – user clusters, web trails, and usage barriers on Germany’s public reporting portal Weisse-Liste.de
Source: BMC Med Inform Decis Mak. 2017 Apr 21;17:48. doi: 10.1186/s12911-017-0440-6 (PMC5399803; doi:10.1186/s12911-017-0440-6)

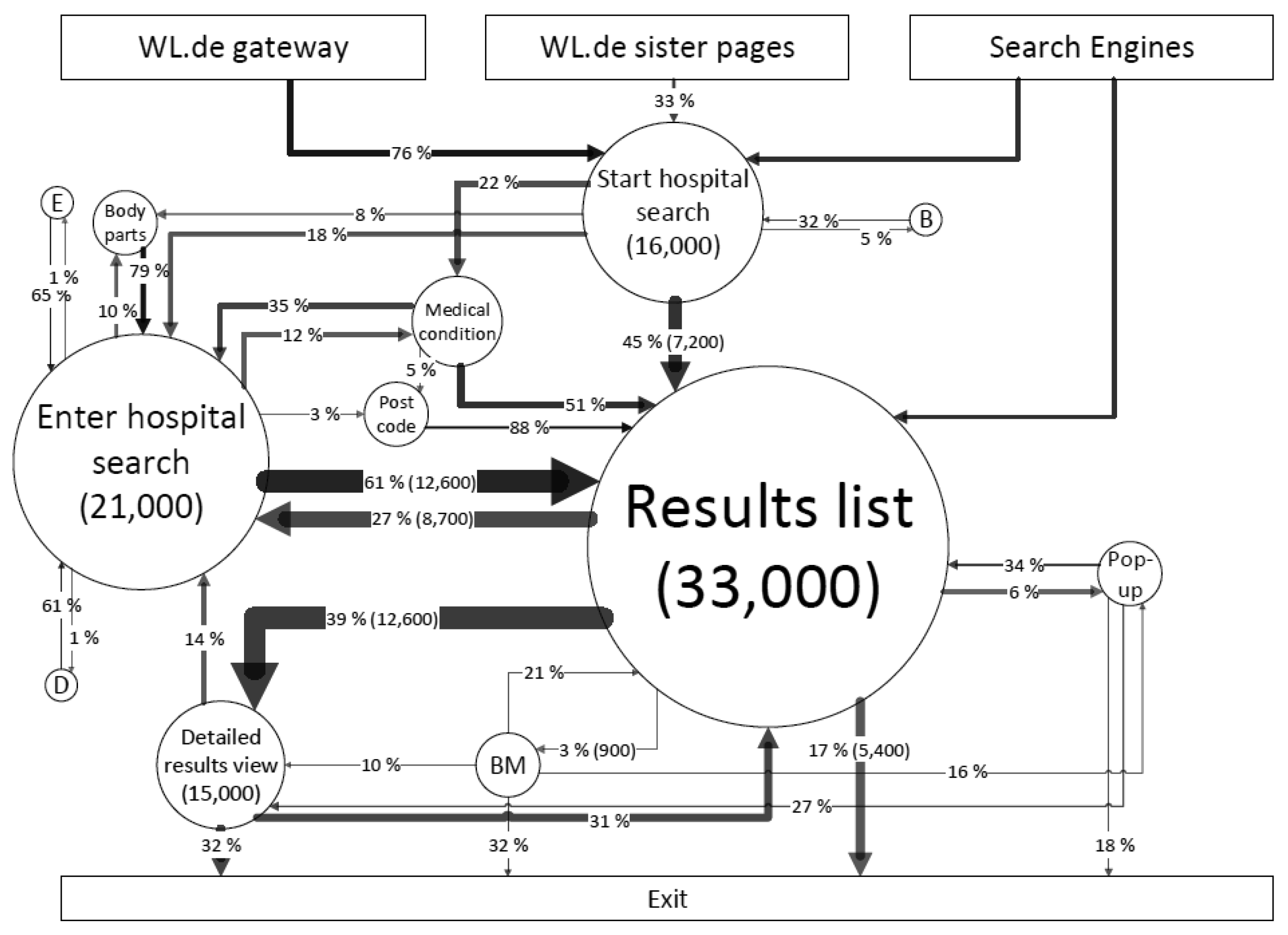

Supplement: Supplementary file 2 — Navigational trail for user group Intensive Work Timers (19% of users). The figure depicts the navigation trail for the specific user subgroup Intensive Work Timers, indicating clicks per topics area (bubble size), absolute number of transitions (arrow width) and share of transitions away from respective topic areas (arrow grayscale). (TIF 875 kb) [file 12911_2017_440_MOESM2_ESM.tif]

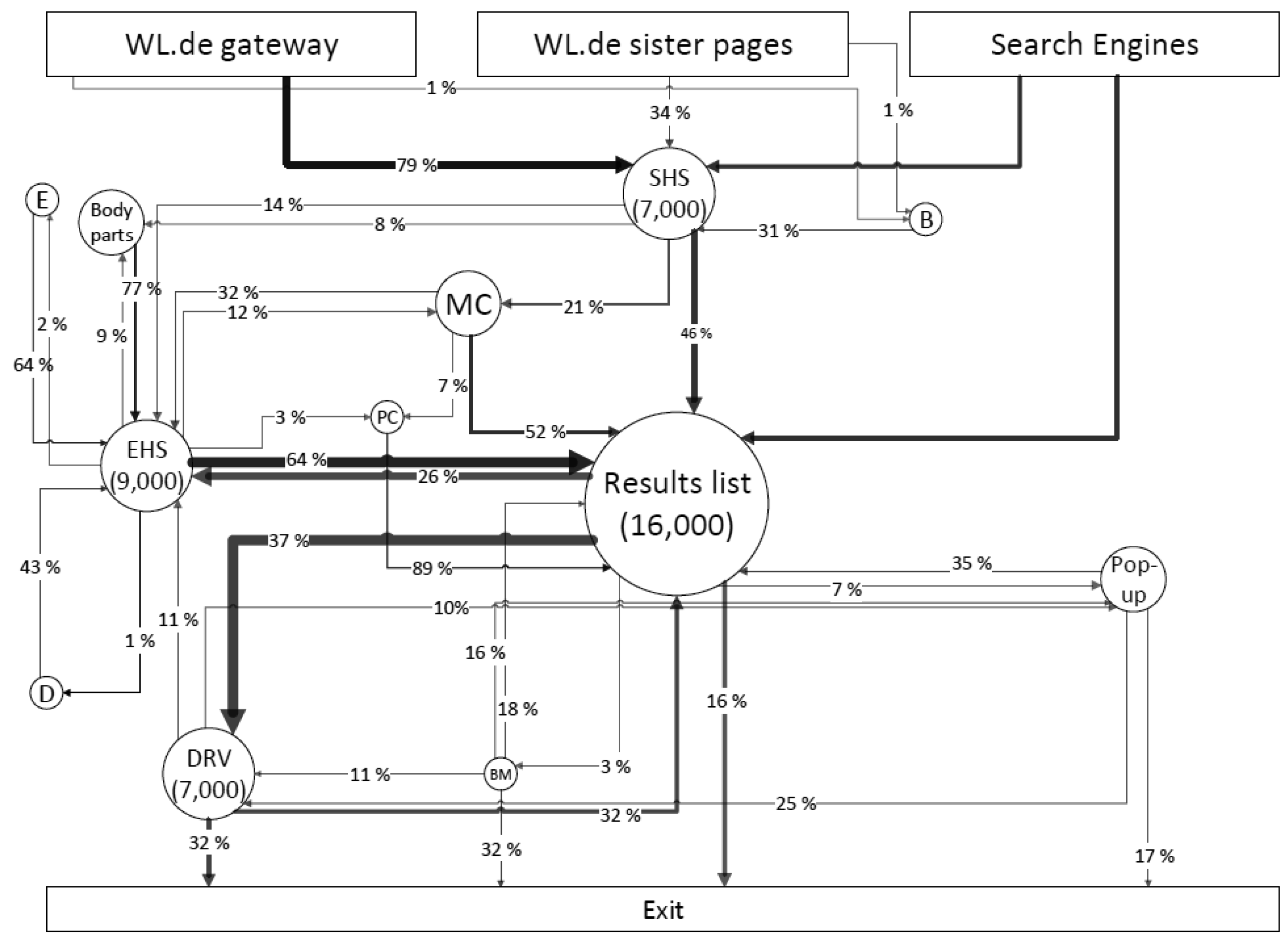

Supplement: Supplementary file 3 — Navigational trail for user group Patient Experts (9% of users). The figure depicts the navigation trail for the specific user subgroup Patient Experts, indicating clicks per topics area (bubble size), absolute number of transitions (arrow width) and share of transitions away from respective topic areas (arrow grayscale). (TIF 726 kb) [file 12911_2017_440_MOESM3_ESM.tif]

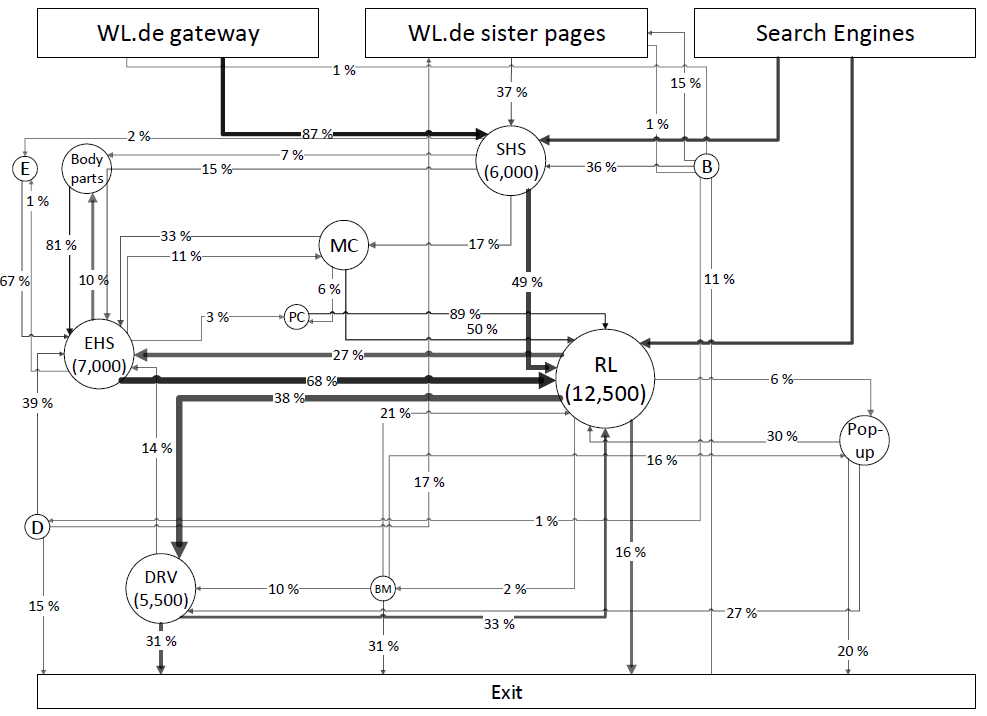

Supplement: Supplementary file 4 — Navigational trail for user group Professionals (7% of users). The figure depicts the navigation trail for the specific user subgroup Professionals, indicating clicks per topics area (bubble size), absolute number of transitions (arrow width) and share of transitions away from respective topic areas (arrow grayscale). (TIF 86 kb) [file 12911_2017_440_MOESM4_ESM.tif]

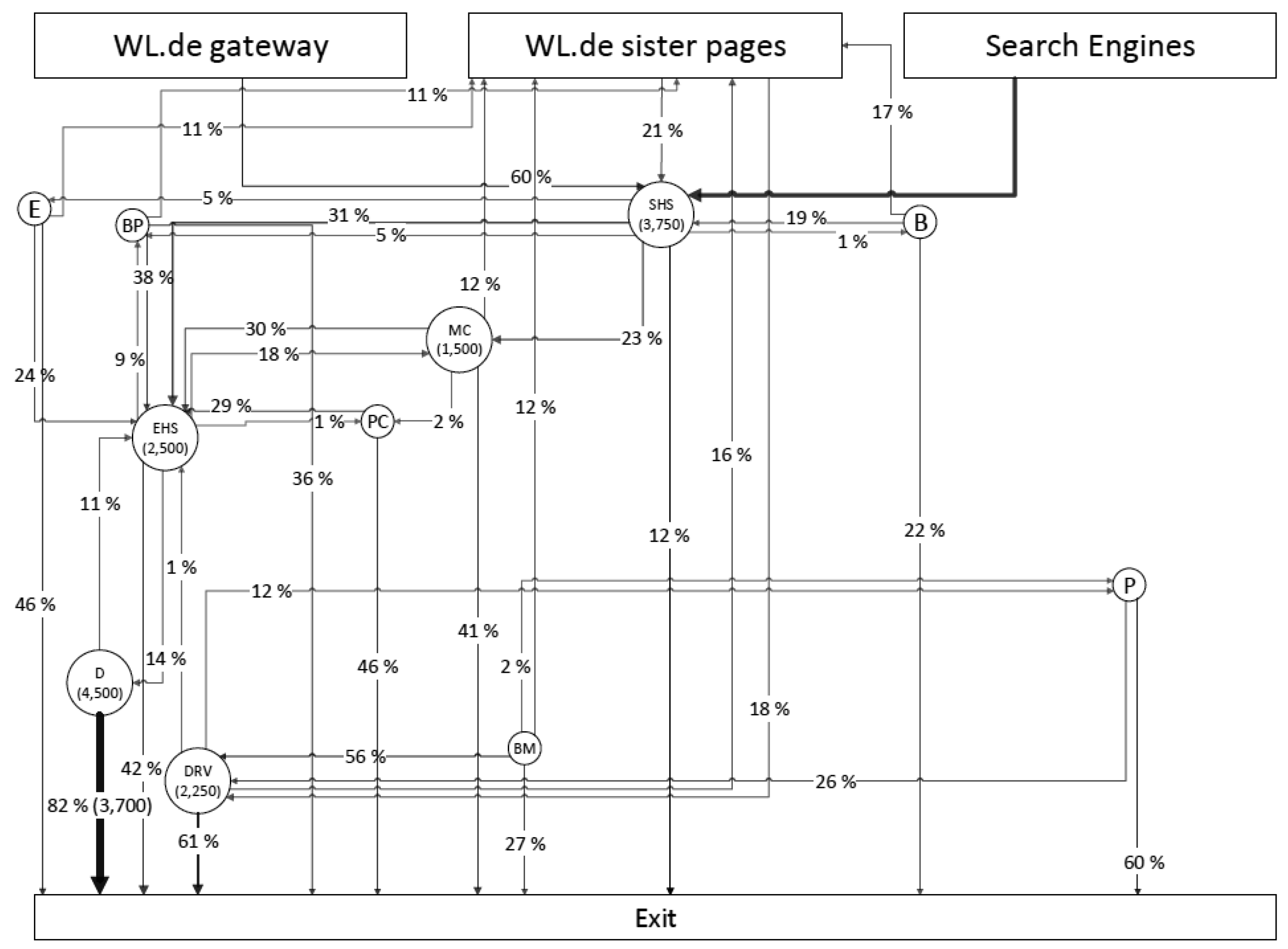

Supplement: Supplementary file 5 — Navigational trail for user group Challenged Aborts (12% of users). Description: The figure depicts the navigation trail for the specific user subgroup Challenged Aborts, indicating clicks per topics area (bubble size), absolute number of transitions (arrow width) and share of transitions away from respective topic areas (arrow grayscale). (TIF 784 kb) [file 12911_2017_440_MOESM5_ESM.tif]
